# Protective effect of vaccination against mumps complications, Czech Republic, 2007–2012

**DOI:** 10.1186/s12889-016-2958-4

**Published:** 2016-04-01

**Authors:** Hana Orlíková, Marek Malý, Pavla Lexová, Helena Šebestová, Radomíra Limberková, Lucie Jurzykowská, Jan Kynčl

**Affiliations:** Department of Infectious Diseases Epidemiology, Centre for Epidemiology and Microbiology, National Institute of Public Health, Šrobárova 48, 10042 Prague, Czech Republic; Department of Biostatistics, National Institute of Public Health, Šrobárova 48, 10042 Prague, Czech Republic; National Reference Laboratory for Measles, Mumps, Rubella and Parvovirus B19, Centre for Epidemiology and Microbiology, National Institute of Public Health, Šrobárova 48, 10042 Prague, Czech Republic; Department of Epidemiology, 3rd Faculty of Medicine, Charles University in Prague, Ruská 87, 10000 Prague, Czech Republic

**Keywords:** Mumps, Mumps complications, Orchitis, Hospitalization, Vaccination, Surveillance

## Abstract

**Background:**

In the Czech Republic, two-dose immunization against mumps achieves 98 % coverage. The routine reporting detects mumps cases, clinical complications, and hospital admissions in unvaccinated but also in vaccinated individuals. Using surveillance data of patients with mumps we assessed the effectiveness of mumps vaccination on mumps clinical complications and hospitalization need. We also investigated the effect of the time since immunization.

**Methods:**

We analysed data on incident mumps cases reported to the Czech national surveillance system in 2007–2012. Using a logistic regression model with adjustment for age, sex, year of onset, and the administrative region, the association between vaccination and the most frequent mumps complications and hospitalization was evaluated. The adjusted odds ratios (ORa) for mumps complications were compared between the vaccinated and non-vaccinated groups, reflecting the vaccine effectiveness (VEa) computed as VEa = (1-ORa)×100. We estimated the risk of mumps complications by the time from vaccination.

**Results:**

From total of 9663 mumps analysed cases 5600 (58 %) occurred in males. The mean age at the disease onset was 17.3, median 16 years. Ninety percent of the study patients had no complications, while 1.6 % developed meningitis, 0.2 % encephalitis, and 0.6 % pancreatitis. Mumps orchitis occurred in 659 (11.8 %) male cases. In total, 1192 (12.3 %) patients required hospitalization. Two doses of vaccine received by 81.8 % cases significantly reduced the risk of hospitalization: ORa 0.29 (95 % CI: 0.24, 0.35). Two doses showed statistically significant VEa 64 % (95 % CI: 46, 79) for meningitis, 93 % (95 % CI: 66, 98) for encephalitis in all cases, and 72 % (95 % CI: 64, 78) for orchitis in males. Vaccine effectiveness for orchitis declined from 81 to 74 % and 56 % in the most affected age groups 10–14, 15–19, and 20–24 years, respectively. Among 7850 two-dose recipients, the rate of complications rose from below 1 to 16 % in categories up to 6 years and 24 and more years after the second dose, respectively.

**Conclusions:**

This study demonstrates a significant preventive effect of two-dose vaccination against mumps complications (orchitis, meningitis, or encephalitis) and hospitalization for mumps. The risk of complications increases with time interval from vaccination. Teenagers and young adults were the most affected age groups.

## Background

Mumps is an acute infection affecting humans and caused by the mumps virus, an RNA virus of the genus *Rubulavirus*, family *Paramyxoviridae* [1]. A third of mumps infections arise without recognised symptoms [2]. Clinically apparent mumps is manifested mainly by the swelling and inflammation of one or both parotid glands. Approximately ten percent of mumps cases develop complications. Epididymo-orchitis is the most frequent complication. Neurological complications as meningitis and less common encephalitis arise, seldom resulting in permanent unilateral deafness. Pancreatitis occurs rather commonly. Oophoritis or mastitis in females is diagnosed less frequently. Other complications as arthritis, myocarditis, nephritis, and polyneuropathy are infrequent. The case fatality rate is very low, with death reported in 1.5 % of mumps cases associated with encephalitis [2].

Complications of mumps worsen and prolong the course of disease, often require hospitalization and thus increase the economic and overall burden of the disease.

Mumps is transmitted by direct contact, droplet spread, or contaminated fomites. The incubation period averages about 16–18 days (range 12–25 days) [1–4]. The spread of the mumps virus can cause mumps outbreaks in susceptible populations. An epidemic of mumps was described by Hippocrates as early as in the 5th century BC [4, 5]. Resurgence and outbreaks of mumps have been reported in many European countries in recent years [6–15].

One mumps virus serotype [3] and based on the phylogenetic analysis, 12 genotypes of the mumps virus were identified (designated A-N, with E and M being unassigned) [16]. Mumps is preventable by immunization. Vaccines containing live attenuated mumps virus have been used worldwide, usually as part of the combined measles, mumps, and rubella vaccines known as MMR. Mumps is a common childhood infection in unimmunized individuals, but in highly vaccinated populations, the disease affects mainly adolescents and young adults [7, 8, 10, 15].

In the Czech Republic, the universal compulsory vaccination against mumps was introduced in 1987, with the measles and mumps vaccine MOPAVAC® and monovalent vaccine PAVIVAC®. The trivalent MMR vaccine TRIVIVAC® was administered since 1995. All previously named vaccines contained the Jeryl-Lynn vaccine strain. Since 2008, the MMR vaccine PRIORIX® comprising the RIT 4385 strain has been used in the vaccination calendar. In addition, on an optional basis, parents may purchase the PRIORIX TETRA® vaccine (against MMR and varicella) for their child, available on the market since 2007.

The national immunization schedule comprises two doses, the first one given from the 15th month of age and the second one given 6–10 months or more after the first dose [17–20]. The vaccination coverage has been evaluated annually by administrative surveys and in the period 2007–2012 ranged from 97.76 to 98.51 % for two doses and from 1.64 to 0.92 % for one dose only [21].

The Czech national surveillance of mumps is comprehensive, countrywide with compulsory reporting. The EU case definition was enshrined in the legislation in the end of 2008. Hospital physicians and general practitioners report all clinical mumps cases to the regional public health authority. Regional epidemiologists bring together the patient's personal, demographic, clinical, laboratory, and epidemiological data, including the vaccination status. Data on each mumps case are entered with the International Classification of Diseases, Tenth revision (ICD10) code into the national electronic reporting system called EPIDAT. Case based data are transferred weekly from the regional to the national level where they are further analysed and outcomes are published monthly [22, 23]. Yearly anonymous case based data are reported to the European Surveillance System (TESSy) database operated by the European Centre for Disease Prevention and Control (ECDC). In the Czech Republic, the data from the national mandatory notification are accessible only for authorised personnel of the public health service including the National Institute of Public Health. For purpose of public health policy data are analysed, results published to inform professionals and public in order to support prevention of infectious diseases and health protection.

Before the introduction of the mumps vaccine to the Czech Republic, mumps epidemics occurred at regular 3–4-year intervals, with tens of thousands of reported cases and a maximum of over 100,000 mumps patients in the 1970s [4, 19, 23]. In the post-vaccination period, mumps incidence sharply declined. However, occasional regional and national outbreaks with several thousand cases occurred in 1995-96, 2005–2006, and 2011–2012 [17–19, 23–25]. Recent two large outbreaks were caused by genotype G mumps virus [18, 26]. In the latest years, mumps affects mainly adolescents, young adults, and school age children and various complications such as orchitis, meningitis, and pancreatitis arise quite frequently not only in unvaccinated but also in immunized individuals.

Despite the comprehensive surveillance and mandatory vaccination strategy with high vaccination coverage being in place in the Czech Republic, mumps cases and mumps complications continue to occur.

Various studies of vaccine effectiveness against mumps complications were conducted in some countries [7, 27, 28]; nevertheless, no relevant analysis to inquire more deeply the situation in the Czech Republic is available.

### Objective

The aim of the study was to assess the effect of vaccination on mumps complications and hospitalization need in mumps cases reported to the Czech national surveillance system during the period 2007–2012. Furthermore, the influence of the time interval from the second dose of vaccine to the development of complications was considered. The present study was conducted in order to assist relevant experts and decision makers in public health.

## Methods

### Data collection

#### Source of data

Data were derived from the electronic Czech national surveillance system EPIDAT.

#### Case definition

A case was defined as any patient with clinical mumps reported to the EPIDAT under the ICD10 code “B26” with onset of the disease in the six-year period between 2007–2012.

#### Inclusion criteria

All patients with clinical mumps whose clear data on age, sex, date and place of disease onset, type of complications of the disease, if any, hospitalization, and vaccination history were available in their records. Records with unclear items were clarified with the respective epidemiologist of the relevant regional public health authority.

#### Exclusion criteria

All cases with missing, ambiguous, or unclear data on vaccination status, disease complications, or hospitalization in their records.

### Analysis of data

#### Statistical analysis

All subjects analysed in this study had mumps. The epidemiological characteristics of the patients with mumps, i.e. age, gender, year of onset, complications, hospitalisation, and vaccination status, were analysed descriptively using absolute and relative frequencies.

To assess the effect of vaccination we compared mumps complications and hospitalization in vaccinated and unvaccinated mumps cases. No controls were sampled from population, no uninfected comparison group was in the study, only mumps cases reported to the surveillance were analysed.

Univariate and multiple logistic regression models were employed to assess the association between vaccination status of the analysed mumps cases and the most frequent mumps complications or hospitalization. The unadjusted odds ratios (OR) and adjusted odds ratios (ORa) were calculated. The latter were adjusted for age, sex, year of onset, and the third level of administrative region. According to the Nomenclature of Units for Territorial Statistics there are 14 administrative regions of the third level in the Czech Republic (NUTS3).

The adjusted vaccine effectiveness (VEa) was computed using the formula VEa = (1-ORa)×100. Vaccine effectiveness against complications and hospitalization is perceived as the proportion of mumps cases who might be protected from complications/hospitalization if previously vaccinated. The adjusted point estimates in relation to the effect of vaccination on complications and hospitalization among unvaccinated and partially and fully vaccinated mumps cases were compared. Vaccine effectiveness for the prevention of orchitis was evaluated in the male population stratified by age groups. The point estimates for OR, ORa, VEa were supplied with 95 % confidence intervals (95 % CI); results with *p* < 0.05 were considered statistically significant.

To consider the possible influence of epidemic period on risk of complications we compared complications occurrence in two periods: the "non-epidemic 2008–2009" with predominantly sporadic cases, and the "epidemic 2011–2012" when the outbreaks occurred. The logistic regression model was used adjusted for number of doses.

Furthermore, in order to estimate the influence of the length of the time interval from the second dose of vaccine to the mumps complications, only the data on mumps cases in two-dose vaccine recipients were analysed. The mumps cases in two-dose vaccine recipients were divided into categories by two-year intervals from the second dose. Additionally, using the multiply logistic regression model, the relationship between the risk of any complication and two potential predictors, the time interval from the second dose and the age at second dose, was assessed. For this purpose, the first three intervals of 0–1.9, 2–3.9, 4–5.9 years from the second dose were combined together and served as the reference time interval category. The odds of complication in each other category was compared to the reference category and characterised by odds ratio.

Finally, the locally weighted scatterplot smoothing (LOWESS) was used to plot a smooth curve characterizing the relationship between probability of complications and time from the second dose.

The statistical analysis was performed in Stata, release 9.2 (StataCorp LP, College Station, TX, USA).

## Results

### Description of the analysed mumps cases

The mumps cases were unevenly distributed within the six-year period; the incidence of the notified mumps cases per 100.000 population was 12.6; 3.9; 3.4; 10.2; 27.5; 37.1 in the respective six years 2007–2012. The highest morbidity was reported during the outbreaks in 2011–2012.

Out of 9898 mumps cases reported in the six-year study period, 9663 met the inclusion criteria and were included in the analysis. The rate of exclusion was 2.6 %.

The description of the 9663 cases analysed is provided in Table 1. Of these cases, 5600 (58 %) were males. The mean age at disease onset was 17.3, median 16 years (range 0–90). The age group 15–19 was the most affected.

**Table 1:** Characteristics of mumps cases, Czech Republic, 2007–2012

	Number of mumps cases	Percent
	9663	100
Year of onset		
2007	1259	13.0
2008	386	4.0
2009	349	3.6
2010	1042	10.8
2011	2840	29.4
2012	3787	39.2
Gender		
Male	5600	58.0
Female	4063	42.0
Age (years)		
Mean	17.3	
Median	16	
Range (min – max)	0–90	
Standard deviation	8.9	
Age group (years)		
0	12	0.1
1–4	242	2.6
5–9	1226	12.7
10–14	2066	21.4
15–19	3599	37.2
20–24	1118	11.6
25–34	957	9.9
35–44	243	2.5
45–54	131	1.4
55–64	52	0.5
65–74	13	0.1
75 +	4	0.0
Vaccination status		
0 doses	1662	17.2
1 dose	71	0.7
2 doses	7907	81.8
3 doses	23	0.2
Complications		
None	8711	90.1
Orchitis^a^	^a^659	^a^11.8
Meningitis	155	1.6
Encephalitis	15	0.2
Pancreatitis	62	0.6
Mastitis	1	0.0
Oophoritis	1	0.0
Other	59	0.6
Hospitalization		
Yes	1192	12.3
No	8471	87.7
Total	9663	100.0

Note: ^a^only males, of 5600 males

Seventeen percent of mumps cases occurred in unvaccinated individuals, 71 (0.7 %) cases in single-dose vaccine recipients, 7907 (81.8 %) cases in two-dose vaccine recipients, and 23 (0.2 %) cases in three-dose vaccine recipients.

Ninety percent of cases had no clinical complications. The most frequent complication was orchitis, reported in 659 (11.8 %) male patients. Meningitis affected 155 (1.6 %), pancreatitis 62 (0.6 %), and encephalitis 15 (0.2 %) of the study patients. A total of 1192 (12.3 %) mumps cases required hospitalization.

### Association between vaccination and hospitalization or clinical complications of mumps

After one dose of the mumps strain containing vaccine, a statistically significant (*p* < 0.05) protective effect against any complication ORa 0.32 (95 % CI: 0.11, 0.91) and for hospitalization was detected ORa 0.32 (95 % CI: 0.13, 0.76) (Table 2).

**Table 2:** Association between vaccination and mumps complications or hospitalization, vaccine effectiveness, Czech Republic, 2007–2012

Type of complication	VACCINATION number of doses^b^	MUMPS number of cases	COMPLICATIONS number of cases (%)	Unadjusted odds ratio (95 % CI)	Adjusted^c^ odds ratio (95 % CI)	*P* value	Adjusted^c^ vaccine effectiveness % (95 % CI)
Any complication	0	1662	430 (25.9)	Ref	Ref		
	1	71	4 (5.6)	0.17 (0.06, 0.47)	0.32 (0.11, 0.91)	0.033	68 (9, 89)
	2	7907	516 (6.5)	0.20 (0.17, 0.23)	0.32 (0.25, 0.39)	<0.001	68 (61, 75)
Orchitis^a^	0	1006	322 (32.0)	Ref	Ref		
	1	33	3 (9.1)	0.21 (0.06, 0.70)	0.34 (0.10, 1.16)	0.086	66 (-16, 10)
	2	4548	333 (7.3)	0.17 (0.14, 0.20)	0.28 (0.22, 0.36)	<0.001	72 (64, 78)
Meningitis	0	1662	66 (4.0)	Ref	Ref		
	1	71	1 (1.4)	0.35 (0.05, 2.52)	0.50 (0.07, 3.80)	0.502	50 (-280, 93)
	2	7907	87 (1.1)	0.27 (0.19, 0.37)	0.34 (0.21, 0.54)	<0.001	64 (46, 79)
Encephalitis	0	1662	12 (0.7)	Ref	Ref		
	1	71	0 (0.0)				
	2	7907	3 (0.0)	0.05 (0.01, 0.19)	0.07 (0.02, 0.34)	0.001	93 (66, 98)
Pancreatitis	0	1662	15 (0.9)	Ref	Ref		
	1	71	0 (0.0)				
	2	7907	47 (0.6)	0.66 (0.37, 1.18)	1.18 (0.49, 2.86)	0.706	-18 (-186, 51)
Hospitalization	0	1662	511 (30.7)	Ref	Ref		
(males + females)	1	71	6 (8.5)	0.21 (0.09, 0.48)	0.32 (0.13, 0.76)	0.010	68 (24, 87)
	2	7907	673 (8.5)	0.21 (0.18, 0.24)	0.29 (0.24, 0.35)	<0.001	71 (65, 76)
Hospitalization	0	1006	417 (41.5)	Ref	Ref		
(males only^a^)	1	33	4 (12.1)	0.19 (0.07, 0.56)	0.30 (0.10, 0.89)	0.030	41 (11, 90)
	2	4548	502 (11.0)	0.18 (0.15, 0.20)	0.26 (0.21, 0.33)	<0.001	74 (67, 79)

*Note*

Ref - reference category (unvaccinated)

^a^only males, of 5587 males

^b^23 cases (13 in males and 10 in females) in three-dose vaccine recipients were not included in the analysis

^c^adjusted for age, gender, year of onset, and region (NUTS3)

In two-dose vaccine recipients, the risk was significantly (*p* < 0.001) reduced (in comparison to unvaccinated) for hospitalization in all patients ORa 0.29 (95 % CI 0.24, 0.35) and in males 0.26 (95 % CI: 0.21, 0.33), reflecting VEa of 71 % (95 % CI: 65, 76) and 74 % (95 % CI: 67, 79), respectively (Table 2).

In two-dose vaccine recipients, the following significant (*p* < 0.001) protective effects VEa were observed: 68 % (95 % CI: 61, 75) for any complication, 64 % (95 % CI: 46, 79) for meningitis, 93 % (95 % CI: 66, 98) for encephalitis in all patients, and 72 % (95 % CI: 64, 78) for orchitis in males. The computed ORa of 1.18 (95 % CI: 0.49, 2.86) for pancreatitis was the only non-significant result (*p* = 0.706), (Table 2).

Orchitis was the most common among adolescents and young males between 15–34 years (Table 3). The proportions of patients with orchitis were the highest in the age groups 25–34 (38.2 %), 35–44 (26.5 %), and 20–24 (18.7 %). Two-dose vaccine effectiveness against orchitis stratified by age groups significantly declined from VEa 81 % (95 % CI: 13, 96) and 74 % (95 % CI: 45, 88) to 56 % (95 % CI: 34, 71) in the age groups 10–14, 15–19, 20–24 years, respectively, and then slightly rose again to VEa 60 % (95 % CI: 27, 78) in the 25–34-year-olds. In small boys and older men, mumps orchitis was reported sporadically (Table 3).

**Table 3:** Association between vaccination and orchitis in males, by age group, Czech Republic, 2007–2012

Age group	MUMPS in males^a^ number of cases	ORCHITIS - number (% of age group) of cases in males	ORCHITIS - number (%) of cases by number of doses			Odds ratio^b^ (95 % CI)		Vaccine effectiveness^b^ % (95 % CI)	
			0 doses	1 dose	2 doses	1 dose	2 doses	1 dose	2 doses
0	9	0 (0.0 %)	0 (0.0 %)	0 (0.0 %)	0 (0.0 %)				
1–4	140	0 (0.0 %)	0 (0.0 %)	0 (0.0 %)	0 (0.0 %)				
5–9	670	4 (0.6 %)	0 (0.0 %)	0 (0.0 %)	4 (100.0 %)				
10–14	1161	30 (2.6 %)	2 (6.7 %)	0 (0.0 %)	28 (93.3 %)		0.19 (0.04, 0.87)		81 (13, 96)
15–19	2088	219 (10.5 %)	10 (4.6 %)	1 (0.5 %)	208 (95.0 %)	0.38 (0.04, 3.61)	0.26 (0.12, 0.55)	62 (-261, 96)	74 (45, 88)
20–24	690	129 (18.7 %)	50 (38.8 %)	1 (0.8 %)	78 (60.5 %)	0.49 (0.06, 4.28)	0.44 (0.29, 0.66)	51 (-328, 94)	56 (34, 71)
25–34	608	232 (38.2 %)	216 (93.1 %)	1 (0.4 %)	15 (6.5 %)	0.49 (0.05, 4.75)	0.40 (0.22, 0.73)	51 (-375, 95)	60 (27, 78)
35–44	117	31 (26.5 %)	31 (100.0 %)	0 (0.0 %)	0 (0.0 %)				
45–54	68	11 (16.2 %)	11 (100.0 %)	0 (0.0 %)	0 (0.0 %)				
55–64	30	1 (3.3 %)	1 (100.0 %)	0 (0.0 %)	0 (0.0 %)				
65+	6	1 (16.7 %)	1 (100.0 %)	0 (0.0 %)	0 (0.0 %)				
Total	5587	658 (11.8 %)	322 (48.9 %)	3 (0.5 %)	333 (50.6 %)	0.21 (0.06, 0.70)	0.17 (0.14, 0.20)	79 (30, 94)	83 (80, 86)

*Note*

^a^13 male three-dose vaccine recipients were not included in this analysis

^b^reference category is 0 doses

Table 4 shows comparison of the risk of complications in epidemic and non-epidemic periods. There was no significant difference between these two periods in occurrence of all complications, orchitis, meningitis and encephalitis. Significantly lower risk in epidemic years was for pancreatitis ORa 0.23 (95 % CI: 0.12, 0.46) and for hospitalization ORa 0.66 (95 % CI: 0.53, 0.81).

**Table 4:** Risk of complications in epidemic years 2011–2012 in comparison to non-epidemic years 2008–2009

	Adjusted^a^ odds ratio	*p* value	95 % confidence interval
Any complication	0.90	0.415	0.70, 1.15
Orchitis	1.02	0.896	0.75, 1.37
Meningitis	1.32	0.390	0.70, 2.47
Encephalitis	2.12	0.470	0.27, 16.34
Meningitis + encephalitis	1.39	0.287	0.76, 2.53
Pancreatitis	0.23	<0.001	0.12, 0.46
Hospitalization (male + female)	0.66	<0.001	0.53, 0.81
Hospitalization (male only)	0.70	0.005	0.54, 0.89

*Note*

^a^odds ratio adjusted for number of doses

### Influence of the time interval from the second dose of vaccine on mumps complications

In total, 7850 mumps cases had a record with clearly documented two-dose immunization. We divided these cases into 12 categories by two-year intervals from the second dose of vaccine and the thirteenth category was 24 years or more from the second dose of vaccine. Only a few cases with complications occurred till six years from the second dose, with a proportion below 1 %. Then the percentage of complications increased to 16.3 % in those vaccinated 24 or more years before the disease onset. The highest numbers of complications occurred in patients immunized approximately 12–20 years before disease onset (Table 5). The mean time interval between the second dose and the disease onset was 12.8 years (standard deviation 4.8). The mean age at the second dose was 2.2 years (standard deviation 0.8) with range from 20 months to 16 years. Age at the second dose turned out to be an independent predictor of complications. In the individuals who received the second dose after four years of age, the odds of complications were significantly (*p* < 0.001) higher.

**Table 5:** Complications in vaccinated mumps patients by time from the second dose, Czech Republic, 2007–2012

Time from the second dose	MUMPS	COMPLICATIONS	COMPLICATIONS	Adjusted^b^ odds ratio	95 % CI	*p* value
in years	number of cases	number of cases	% of mumps cases with complications			
0–1.9	106	1	0.94	Ref^a^		
2–3.9	287	1	0.35			
4–5.9	435	4	0.92			
6–7.9	650	11	1.69	2.49	0.91, 6.79	0.074
8–9.9	652	10	1.53	2.21	0.79, 6.13	0.126
10–11.9	865	27	3.12	4.70	1.92, 11.47	0.001
12–13.9	1268	79	6.23	10.3	4.33, 23.21	<0.001
14–15.9	1584	128	8.08	13.59	5.93, 31.13	<0.001
16–17.9	1129	136	12.05	21.28	9.29, 48.76	<0.001
18–19.9	447	52	11.63	20.14	8.53, 47.55	<0.001
20–21.9	240	31	12.92	22.97	9.40, 56.11	<0.001
22–23.9	144	21	14.58	26.70	10.51, 67.87	<0.001
24 and later	43	7	16.28	30.41	9.67, 95.59	<0.001
Total	7850	508	6.47			

*Note*

^a^reference category 0–5.9 compounded from three categories: 0–1.9, 2–3.9, 4–5.9 years

^b^odds ratio adjusted for age at the 2nd dose

**Fig. 1:**
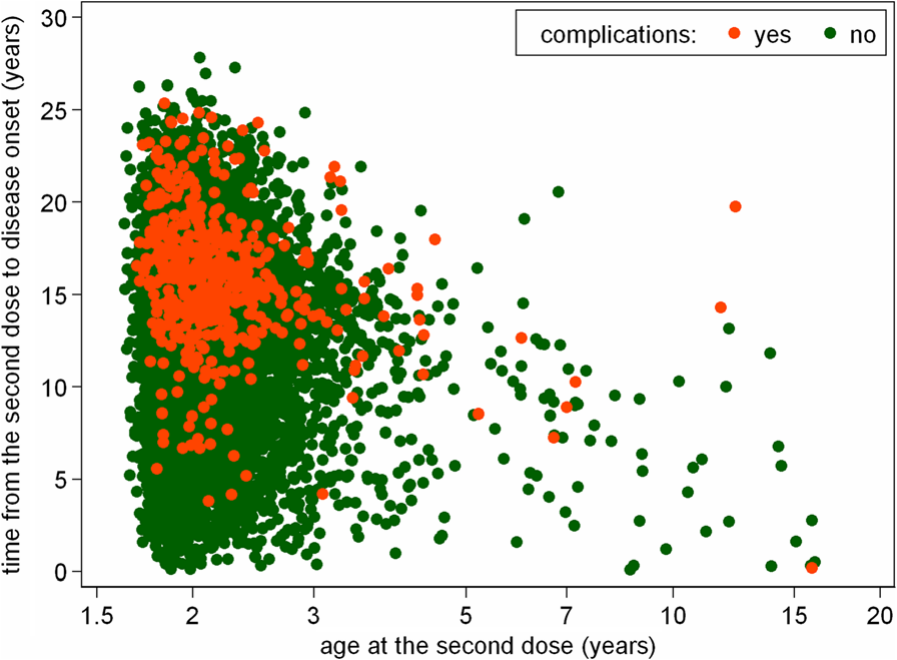
Relationship between time from the second vaccine dose to mumps onset and age at the second dose, by complications

Figure 1 shows the relationship between time interval from the second dose to disease onset and the age at the second dose with regards to the occurrence of complications. Majority of cases were vaccinated to 3 years of age. Only 291 patients received the second dose later. Those who were vaccinated later tended to contract the disease earlier after the completion of the vaccination compared to those vaccinated properly according to the recommended schedule.

**Fig. 2:**
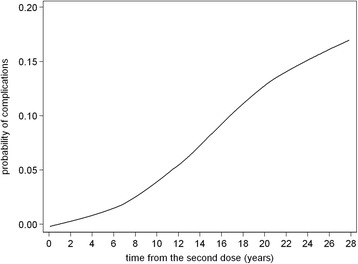
Relationship between probability of complications and time from the second dose

Figure 2 shows how the probability of complications increases with time interval from the second dose (as a continuous variable).

## Discussion

This study was the first investigation of the vaccine effectiveness against clinical complications of mumps and need for hospitalization in the Czech Republic, based on the national surveillance data.

A statistically significant effectiveness of the mumps vaccination on the prevention of orchitis, meningitis, encephalitis, and hospitalization was documented in this study. The most frequent clinical complication was orchitis. The most afflicted age groups were teenagers, adolescents, and young adults, similarly to some other studies [7, 10]. The findings of the risk of orchitis growing with age of male corresponds with the reference, in which the older age at infection is associated with a higher risk of certain complications, particularly orchitis [2].

The point estimates in this study are slightly different from the results reported by Dutch and British authors [7, 27]. Anyway, all three studies have proven the protective effect of mumps vaccination with two doses. In two-dose vaccine recipients the risk was reduced for orchitis (ORa 0.64, 0.26, 0.28) and for hospitalization (ORa 0.45, 0.18, 0.29) in England and Wales, the Netherlands, and the Czech Republic, respectively.

In addition, a significant protective effect against clinical complications and hospitalization was observed among single-dose vaccine recipients in this study. The national immunization calendar prescribes two MMR doses. The second dose of MMR vaccine is not a booster, but rather is given as another individual dose. In this study, there were small numbers of mumps cases and complications among the single-dose recipients. Even higher protective effect against all complications has been reported in the Netherlands [7] in single-dose and two-dose vaccine recipients, with ORa of 0.29 (95 % CI: 0.14, 0.62) and 0.24 (95 % CI: 0.14, 0.39), respectively. Therefore, the general importance of mumps vaccination should be emphasized. Each single dose of mumps vaccine can contribute to the protection against mumps complications.

To see if the outbreak period can somehow affect the occurrence of particular complications we compared epidemic and non-epidemic years. No substantial differences were seen in the occurrence of severe complications with exception of pancreatitis. The odds of hospitalization was lower during the epidemic period, this might be due to the limited capacities in the hospital health care settings.

Another important outcome of this investigation was the growing risk and thus decreasing protection against mumps complications with time from the second vaccine dose as prescribed in the national immunization schedule. Nevertheless, the risk still remained lower in comparison with the unvaccinated. Several patients who received the dose during the incubation period, mostly within the immunization campaign in response to the 2011 mumps outbreak in the Ústí nad Labem Region [24], developed mumps a few days later.

The patients immunized later than at four years of age had higher odds of complications. Unfortunately, routine surveillance data do not allow a clear interpretation of this finding. Various factors, e.g. an underlying or chronic disease, may have played a role in delayed vaccination [29].

When comparing the results of this study with those of the sero-epidemiological survey conducted in the population of the Czech Republic in 2013 [30], a similar downward trend in the specific antibody protection against mumps was revealed by the serosurvey in teenagers from the highly vaccinated general population. Thus the results of this study might indirectly support the probable impact of the waning immunity on increase in mumps cases and complications with time from the second vaccine dose in the vaccinated population. The issues of the waning immunity or secondary vaccine failure [31] and growing risk of developing mumps with increasing time after vaccination have been addressed [32, 33]. Due to the secondary vaccine failure after the previous vaccination the decreased or insufficient specific antibody level is unable to protect an individual infected by circulating wild strain of virus from disease development. Nevertheless, in the present study no laboratory data were analysed and the immunity status thus assumption of waning immunity couldn’t be validate directly. No uninfected comparison group was in the study. We also have to admit that orchitis as the most frequent complications contributed essentially to the number and proportion of all complications. Majority of the orchitis cases were among adolescents and young adults, and in the older age the risk of orchitis is higher [2]. It should be mentionded as a theoretical possibility of an intrinsic bias. Additionally, it was not possible to differentiate between primary and secondary vaccine failure in particular vaccinated cases included in this study.

This study does not investigate the impact of particular vaccine types on mumps complications. Vaccine effectiveness is not compared between different vaccine virus strains. Generally, both vaccine strains used in the Czech Republic (Jeryl Lynn and RIT 4385) are derived from genotype A [19]. Both vaccine strains are considered to be effective against the live mumps virus of genotype G, identified in some outbreak cases in 2006 and 2012 [26].

The authors are aware of the study limitations. Where appropriate, correction was applied for possible biases in particular steps of the present research protocol. To minimize the selection bias the data were collected directly from the national database. In the analyses, the multiple logistic regression adjusting for the important predictors (age, gender, year of onset, NUTS3 regions) was used to correct for possible information biases. The fact that the data originate from the routine surveillance system was taken into account. In the process of data collection and data cleaning, great efforts were made to clarify unclear, ambiguous, or missing data to minimize loss of records. Despite the notification is comprehensive, mandatory, enshrined in law [34, 35], and stable within the recent decades, the passive surveillance system might be subject to underreporting.

Another limitation might be that all reported mumps cases were included in this study regardless of their classification. The majority of cases were laboratory confirmed or epidemiologically linked to a confirmed case. Suspected cases are reported rarely based on typical clinical symptoms. Therefore, the notified mumps cases are unlikely to have been subject to misdiagnosis. The study relies on careful appraisal of each notified mumps case by the reporting physician and epidemiologist who performed epidemiological investigation of all reported cases.

This research might contribute to a better understanding of why mumps and mumps outbreaks occur in the Czech population with a high vaccination coverage. Recently, the Czech population has not been notably exposed to the natural booster by the wild virus. Supposing some gaps in the protection, herd immunity might drop to a certain threshold and then the wider spread of mumps might hit susceptible individuals. In the present investigation, the highest numbers of mumps cases and complications were in the age group 15–19 years.

The current Czech two-dose vaccination schedule is completed in children at the age of 21 to 25 months. The present results show that mumps and the clinical complications of mumps are quite rare during approx. 6-year period after the second dose. The epidemiological situation where mumps and complications affect predominantly teenagers and young adults evoke thinking about the need for updating the immunization calendar. The postponement of the second dose to an older age could be discussed. However, to determine the optimal age and adjust the schedule will require further investigation, taking into account laboratory and serological survey results. The epidemiological situation of measles and rubella should also be considered when speaking about the combined MMR vaccine routinely used in the Czech immunization practice.

## Conclusions

Results of the present analysis confirm the positive preventive effect of vaccination on mumps complications in the context of the epidemiological situation of mumps, vaccination policy, and surveillance system in the Czech Republic.

The risk of clinical complications and hospitalization is lower in the vaccinated than in the unvaccinated patients with mumps. Immunization with two doses of mumps strain containing vaccines significantly reduces the risk of encephalitis, meningitis, orchitis and hospitalization. The risk of complications is not influenced substantially by the epidemic period. Orchitis, the most frequent complication of mumps, affects mainly teen-age, adolescent and young adult males. Within these age groups the vaccine effectiveness for orchitis declines with the growing age.

In two-dose recipients the risk of all complications increases and the protection declines with time interval since the previous vaccination. Additional studies would be needed to investigate the serological background of findings in the present study. To decrease the burden of mumps and mumps complications in the most affected age groups the adjustment of the vaccination schedule could be discussed. Further studies are required to determine the best approach to immunization in compliance with the current needs.

## Abbreviations

CEM: Centre for Epidemiology and Microbiology
CI: confidence interval
ECDC: European Centre for Disease Prevention and Control
EU: European Union
ICD10: the 10th revision of the International Statistical Classification of Diseases and Related Health Problems
MMR: measles, mumps, rubella
NUTS: Nomenclature of Units for Territorial Statistics (NUTS3 refers to the third level of regions; there are 14 NUTS3 regions in the Czech Republic)
OR: odds ratio
ORa: adjusted odds ratio
VEa: adjusted vaccine effectiveness

## References

[1] Plotkin SA, Rubin SA: Mumps vaccine. In: Plotkin SA, Orenstein WA, Offit PA. Vaccines. 5th ed. Philadelphia: W.B. Saunders Elsevier Inc.; 2008. p. 435-465

[2] Hviid A, Rubin S, Mühlemann K (2008). Mumps. Lancet.

[3] McLean HQ, Hickman CJ, Seward JF (2010). World Health Organization, Department of Immunization, Vaccines and Biologicals. The Immunological Basis for Immunization Series. Module 16: Mumps.

[4] Beran J, Havlik J, Vonka V: Parotitis epidemica – mumps. In: Vaccination – past, present, future. Galen; 2005. p. 63-65. [in Czech]

[5] Tsoucalas G, Laios K, Karamanou M, Androutsos G (2013). The Thasian epidemic of mumps during the 5th century BC. Infez Med.

[6] Kay D, Roche M, Atkinson J, Lamden K, Vivancos R (2011). Mumps outbreaks in four universities in the North West of England: prevention, detection and response. Vaccine.

[7] Sane J, Gouma S, Koopmans M, de Melker H, Swaan C, van Binnendijk R, Hahne S (2014). Epidemic of mumps among vaccinated persons, The Netherlands, 2009–2012. Emerg Infect Dis.

[8] Stein-Zamir C, Shoob H, Abramson N, Tallen-Gozani E, Sokolov I, Zentner G. Mumps outbreak in Jerusalem affecting mainly male adolescents. Euro Surveill. 2009;14(50). 20070937

[9] St-Martin G, Knudsen LK, Engsig FN, Panum I, Andersen PH, Rønn J, Fonager J, Fischer TK (2014). Mumps resurgence in Denmark. J Clin Virol.

[10] Otto W, Mankertz A, Santibanez S, Saygili H, Wenzel J, Jilg W, Wieland W, Borgmann S. Ongoing outbreak of mumps affecting adolescents and young adults in Bavaria, Germany, August to October 2010. Euro Surveill. 2010;15(50). 21172171

[11] Hukic M, Hajdarpasic A, Ravlija J, Ler Z, Baljic R, Dedeic Ljubovic A, Moro A, Salimovic-Besic I, Sausy A, Muller CP, Hubschen JM. Mumps outbreak in the Federation of Bosnia and Herzegovina with large cohorts of susceptibles and genetically diverse strains of genotype G, Bosnia and Herzegovina, December 2010 to September 2012. Euro Surveill. 2014;19(33). 10.2807/1560-7917.es2014.19.33.20879 25166347

[12] Walker J, Huc S, Sinka K, Tissington A, Oates K. Ongoing outbreak of mumps infection in Oban, Scotland, November 2010 to January 2011. Euro Surveill. 2011;16(8). 21371413

[13] Rajcevic S, Seguljev Z, Petrovic V, Medic S, Nedelijkovic J, Milosevic V, Turo L, Ristic M. Ongoing mumps outbreak in Novi Sad, the autonomous province of Vojvodina, Serbia, January to April 2012. Euro Surveill. 2012;17(19). 22607963

[14] Braeye T, Linina I, De Roy R, Hutse V, Wauters M, Cox P, Mak R (2014). Mumps increase in Flanders, Belgium, 2012–2013: results from temporary mandatory notification and a cohort study among university students. Vaccine.

[15] Vareil MO, Rouibi G, Kassab S, Soula V, Duffau P, Lafon ME, Neau D, Cazanave C (2014). Epidemic of complicated mumps in previously vaccinated young adults in the South-West of France. Med Mal Infect.

[16] WHO. Mumps virus nomenclature update: 2012. Wkly Epidemiol Rec. 2012;87(22):217–24 24340404

[17] Boxall N, Kubinyova M, Prikazsky V, Benes C, Castkova J: An increase in the number of mumps cases in the Czech Republic, 2005–2006. Euro Surveill. 2008;13(16). 18768117

[18] Kubinyiova M, Benes C, Prikazsky V, Roubalova K, Castkova J. Mumps vaccination in the Czech Republic. Euro Surveill. 2008;13(27). 18761932

[19] Lexova P, Limberkova R, Castkova J, Kyncl J (2013). Increased incidence of mumps in the Czech Republic in the years 2011 and 2012. Acta Virol..

[20] Decree No. 537/2006 Coll. on Vaccination against Infectious Diseases. [in Czech]

[21] Dlhy J. Administrative estimate of vaccination coverage in the Czech Republic by December, 2010. Zpravy CEM (SZU, Praha), 2012;21(3):92–97. [in Czech]

[22] Zpravy Centra Epidemiologie a Mikrobiologie, SZU Praha [in Czech]

[23] Infekce v CR – EPIDAT. National Institute of Public Health. http://www.szu.cz/publikace/data/infekce-v-cr. Accessed 29 July 2015. [in Czech]

[24] Trmal J, Koci J, Simunkova L, Storkanova O, Trmalova Z: Mumps outbreak in the Usti administrative region. Zpravy CEM (SZU, Praha). 2011;20(6):219–223. [in Czech]

[25] Stepanova V, Pliskova L, Kosina P, Splino M, Forstl M, Bolahovska R, Dlhy J, Chrzova M (2006). Mumps – a reemerging infection? The current incidence of mumps in the East Bohemian region in the Czech Republic. Epidemiol Mikrobiol Imunol.

[26] Limberkova R, Lexova P (2014). Genotyping results, laboratory diagnosis, and epidemiology of the mumps virus circulating in the Czech Republic in 2012 Goal. Epidemiol Mikrobiol Imunol.

[27] Yung CF, Andrews N, Bukasa A, Brown KE, Ramsay M (2011). Mumps complications and effects of mumps vaccination, England and Wales, 2002–2006. Emerg Infect Dis.

[28] Hahne S, Whelan J, van Binnendijk R, Swaan C, Fanoy E, Boot H, de Melker H (2012). Mumps vaccine effectiveness against orchitis. Emerg Infect Dis.

[29] van der Meer H, Kimpen JL (1996). Insufficient vaccination status of children with a chronic disease. Ned Tijdschr Geneeskd.

[30] Public Health Institute in Ostrava, Public Health Institute in Usti nad Labem. Mumps. Multi-purpose serological survey (measles, mumps, pertussis, viral hepatitis B), 2013, Czech Republic. Zpravy CEM (SZU, Praha), 2014; 23: Suppl 1: 36–55. [in Czech]

[31] Park DW, Nam MH, Kim JY, Kim HJ, Sohn JW, Cho Y, Song KJ, Kim MJ (2007). Mumps outbreak in a highly vaccinated school population: assessment of secondary vaccine failure using IgG avidity measurements. Vaccine.

[32] Cortese MM, Jordan HT, Curns AT, Quinlan PA, Ens KA, Denning PM, Dayan GH (2008). Mumps vaccine performance among university students during a mumps outbreak. Clin Infect Dis.

[33] Castilla J, Garcia Cenoz M, Arriazu M, Fernández-Alonso M, Martinez-Artola V, Etxeberria J, Irisarri F, Barricarte A (2009). Effectiveness of Jeryl Lynn-containing vaccine in Spanish children. Vaccine.

[34] Act No. 258/2000 Coll. on Public Health Protection. [in Czech]

[35] Decree No. 473/2008 Coll. on Epidemiological Surveillance System for Selected Infections. [in Czech]

